# Constitutive Model and High Temperature Rheological Behavior of PEEK

**DOI:** 10.3390/polym18202461

**Published:** 2026-10-09

**Authors:** Jiaxin Deng, Chun Zhang, Xiangming Chen, Tiesong Zhang, Xitao Zheng

**Affiliations:** 1 School of Aeronautics, Northwestern Polytechnical University, Xi’an 710072, China; 2 National Key Laboratory of Strength and Structural Integrity, Xi’an 710072, China; 3 National Key Laboratory for Strength and Structural Integrity, Aircraft Strength Research Institute of China, Xi’an 710072, China

**Keywords:** poly-ether-ether-ketone, flow stress, constitutive model, strain rate sensitivity, temperature sensitivity, steady-rate flow

## Abstract

Accurately characterizing the mechanical behavior of polyether ether ketone (PEEK) under low strain rate conditions and over a wide temperature range is essential for predicting the mechanical performance of PEEK and PEEK-based composites. In this study, PEEK grade ZYPEEK^®^-330G was used. Specimens were prepared by injection molding. Quasi-static uniaxial tensile tests were conducted at temperatures of 20, 50, 80, 110, and 140 °C and strain rates from 10^−4^ to 10^−1^ s^−1^, with three replicate specimens for each condition. The high-temperature rheological behavior and flow stress below glass transition temperature (Tg ≈ 143 °C) were studied based on the free volume theory. Results indicate that with the increasing of temperature and the decreasing of strain rate, the yield strength, flow stress and elastic modulus of PEEK decrease, while the ductility and toughness are enhanced. The strain rate sensitivity index demonstrates a transition trend from non-Newtonian flow to Newtonian flow below Tg. A seven-parameter modified constitutive model considering both strain rate and temperature effects is proposed to accurately characterize the tensile mechanical response of PEEK before yielding. Model parameters were determined from the true stress–strain data at 20, 80, 110, and 140 °C, and the model was independently validated against the 50 °C data not used for fitting, with a prediction error of less than 3%. The model is limited to the pre-yield regime, the quasi-static strain rate range of 10^−4^ to 10^−1^ s^−1^, and temperatures below Tg. These findings demonstrate that the tensile response of PEEK is sensitive to both temperature and strain rate, and this constitutive model provides a foundation for predicting the tensile behavior of PEEK under complex environmental conditions.

## 1. Introduction

**Figure 1:**
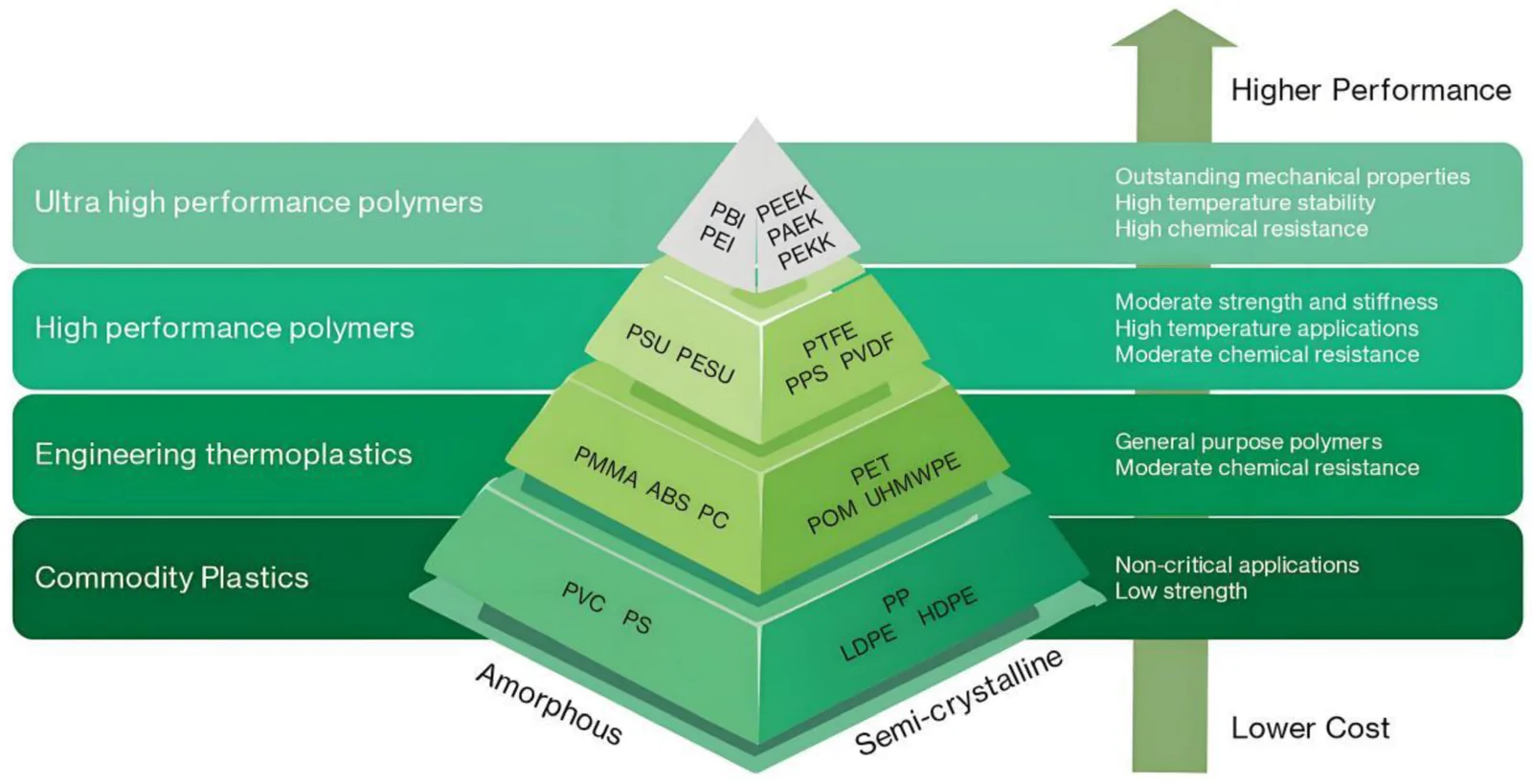
Hierarchical structure of amorphous and semi-crystalline thermoplastics [1].

PEEK is a semi-crystalline thermoplastic high-performance specialty engineering material, as shown in Figure 1. PEEK exhibits excellent thermal stability, chemical resistance, and significant mechanical characteristics including high stiffness, high strength, wear resistance, and high-temperature resistance, which are retained at elevated temperatures.

These attributes have established PEEK and its composites [2,3,4,5] as critical materials in aerospace, automotive, and medical applications [6,7,8,9]. Therefore, accurately characterizing the mechanical properties and proposing the constitutive model of PEEK are important for expanding applications in a wider field [10].

The mechanical properties of PEEK have been extensively studied in the last few decades. A significant body of work has focused on its behavior across wide ranges of strain rates and temperatures. For instance, El-Qoubaa and Othman [11,12,13] systematically investigated the tensile mechanical behavior of PEEK across a broad range of strain rates (0.001 to 1000 s^−1^). D. Barba et al. [14] studied the uniaxial tension tests of PEEK at temperatures from 20 to 240 °C, and strain rates from 0.0001 to 0.1 s^−1^. Hamdan and Swallowe [15] investigated the compressive flow stress of PEEK over a wide range of strain rates (10^−3^ to 10^3^ s^−1^) and temperatures (20 to 200 °C). Tang et al. [16] investigate the mechanical properties and failure mechanisms at various temperatures (−30 to 100 °C) and strain rates (10^−3^ to 10^3^ s^−1^). Chen et al. [17] investigated the tensile properties of PEEK and two kinds of short-fiber (carbon fiber and glass fiber) reinforced PEEK under uniaxial tensile tests of different strain rates (10^−3^ to 10^3^ s^−1^). Cady et al. [18] studied the flow stress and strain behaviors of PEEK at different temperatures and strain rates. Rae et al. [19] studied the compressive behaviors of PEEK under strain rates ranging from 10^−4^ to 3000 s^−1^ and temperatures ranging from −85 °C to 200 °C, and tensile behaviors under strain rates ranging from 2.7 × 10^−5^ to 1.9 × 10^−2^ s^−1^ and temperatures ranging from −50 °C to 150 °C. The tensile and compressive responses have been systematically investigated from quasi-static to high strain rates (up to 10^3^ s^−1^) and across temperatures ranging from sub-ambient to near-melting points. These studies have collectively established that PEEK exhibits strong sensitivity to both strain rate and temperature, with yield stress and flow behavior being profoundly influenced.

More recently, attention has shifted towards understanding the high-temperature rheological behavior of PEEK, particularly in its melt state. Wang et al. [20] used differential scanning calorimetry (DSC) and rotational rheology testing to investigate the rheological properties of PEEK and PEEK-reinforced composites. Croshaw et al. [21] investigated dynamic rheological behavior of PEEK in the melt state. Bangarusampath et al. [22] measured the rheotens of PEEK-reinforced composites and calculated the apparent viscosity over a wide range of elongational rates. However, despite this progress, current research on the high-temperature rheological behavior of PEEK has predominantly focused on the molten state, whereas its solid-state rheological behavior below and near the glass transition temperature (Tg), including steady-state flow stress, strain rate sensitivity, and apparent viscosity, has received considerably less attention. Unlike the melt-state behavior dominated by chain entanglements, the rheology in this transitional zone is governed by different mechanisms, such as free volume evolution. The rheological response in this temperature range, particularly its coupling mechanism with strain rate, remains insufficiently explored. Within this specific thermal region, where the material undergoes a transition from the glassy to the rubbery state, the free volume theory provides a robust framework for understanding the underlying rheological behavior.

Several constitutive models based on experimental results have been developed to characterize the mechanical response of polymer. Empirical models like the Johnson–Cook (J-C) and the Zhu–Wang–Tang (ZWT) have been widely adopted and subsequently modified for polymeric materials, including PEEK. The Johnson–Cook model, as a traditional empirical constitutive model [23], has been widely used as the foundation for most current nonlinear viscoelastic constitutive models, which have been developed through modifications and improvements to the original J-C framework. Chen et al. [24,25] employed the Johnson–Cook constitutive model to predict the compressive and tensile flow stress of PEEK, and further modified the Johnson–Cook model. Zhang et al. [26] proposed a segmented Johnson–Cook constitutive model for polymeric materials such as PEEK under varying mechanical states, which enables more precisely to characterize the mechanical behavior of PEEK under moderate to elevated temperatures. Wang et al. [27] developed an modified Zhu–Wang–Tang (ZWT) constitutive model based on the original ZWT, incorporating both strain rate and temperature effects to predict the mechanical impact response of PMMA. Yao et al. [28] created a constitutive model combining the Weibull distribution with the ZWT constitutive model. By substituting temperature-dependent interpolation functions into the ZWT constitutive equations, a modified ZWT constitutive model was obtained.

Furthermore, sophisticated viscoelastic, viscoelastoplastic, and fractional models have been developed to describe specific phenomena like pre-yield behavior and creep. Zheng et al. [29] investigated numerous constitutive models, DSGZ was employed to characterize the deformation behavior of PEEK at elevated temperatures. Xu et al. [30] developed a corporative viscoelastoplastic constitutive model to characterize the dynamic response of semi-crystalline polymers based on the mechanical response. Based on an elastic–viscoplastic rheological approach, Richeton et al. [31,32] developed a three-dimensional constitutive model to describe the mechanical response of polymers over a wide range of temperatures and strain rates. According to the uniaxial extension and compression tests, Li et al. [33] fitted a hyperelastic constitutive model. Cai et al. [34] developed a fractional rheological model to describe creep and relaxation behaviors of polymer. Chang et al. [35] investigated the uniaxial tensile response of PEEK during the pre-necking phase and developed a phenomenological nonlinear viscoelastic constitutive model to accurately characterize the tensile mechanical responses under low strain rates and wide temperature ranges. Cao et al. [36] proposed a viscoelastic constitutive model with a nonlinear spring and a nonlinear Maxwell element to describe the deformation behavior of polycarbonate before yielding at different strain rate and temperature. Drozdov and Christiansen [37] proposed constitutive models for the thermo-mechanical behavior of PEEK.

Despite these advances, existing nonlinear viscoelastic models for PEEK still face challenges in simultaneously capturing the coupled effects of strain rate and temperature in the pre-yield regime with a compact parameter set. In this study, a seven-parameter nonlinear viscoelastic constitutive model is proposed. It differs from existing models in three main aspects. First, it combines a fourth-order nonlinear spring with a nonlinear Maxwell unit, so that the nonlinear elastic and viscous responses are represented separately with clear physical meaning. Second, the relaxation time of the Maxwell unit is defined as a power-law function of strain rate, which enables the model to capture strain rate sensitivity without adding empirical fitting parameters. Third, six of the seven parameters are expressed as temperature-dependent functions via polynomial interpolation, allowing continuous prediction over the tested temperature range rather than only at discrete temperatures. The model is limited to the pre-yield regime and provides a compact, physically interpretable, and accurate description of the tensile response of PEEK under low strain rates and temperatures below *T_g_*.

This paper aims to analyze the mechanical properties of PEEK materials by conducting quasi-static uniaxial tensile tests, and considers the effect of temperatures ranging from 20 °C to 140 °C and strain rates ranging from 10^−4^ to 10^−1^ s^−1^ on the experimental tensile response. The experiment investigates high-temperature rheological behavior under steady-state conditions and the sensitivity of strain rates and temperatures. The rheological properties was studied experimentally based on the free volume theory below glass transition temperature. The relationship between the flow stress and strain rates was further investigated, as well as the relationship of the strain rate sensitivity index and apparent viscosity with strain rates. Then, through the true data, a seven-parameter phenomenological constitutive model that can characterize the mechanical response of PEEK materials within the strain rate and temperature rate studied in this article is proposed. The phenomenological constitutive model proposed in this paper incorporates a suitable temperature interpolation function to characterize the mechanical behavior of PEEK, enabling it to simultaneously account for the effects of strain rate and a wide range of test temperatures. The model accurately captures the mechanical response and nonlinear viscoelastic behavior of PEEK under low strain rates and over a broad temperature range (within which a finer subdivision of temperature intervals is employed), with the prediction error controlled within 3%.

## 2. Tensile Test of PEEK

### 2.1. Quasi-Static Uniaxial Tensile Test of PEEK Materials

**Figure 2:**
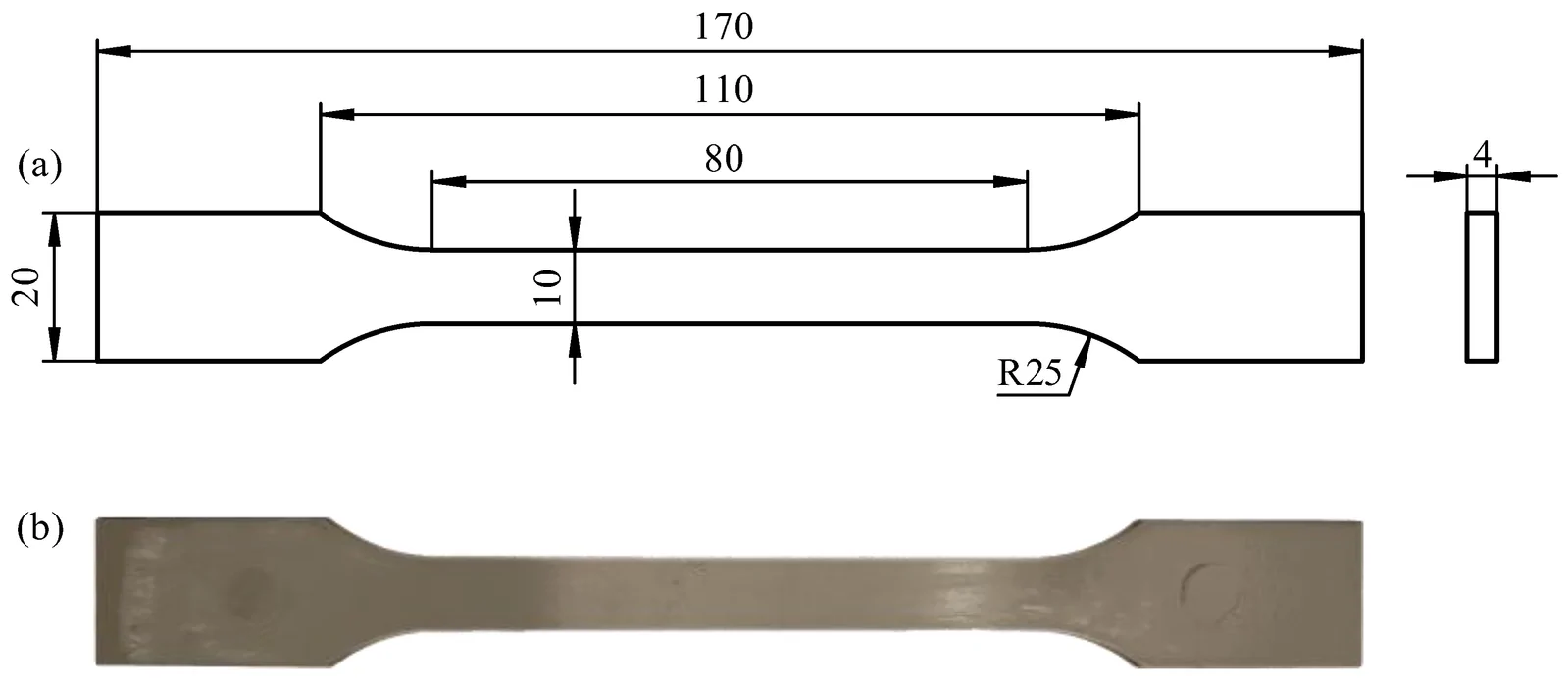
(**a**) geometric shape and dimensions, unit: mm, (**b**) physical photo.

The PEEK grade used was ZYPEEK^®^-330G (Ningbo Shuxiang Aerospace Composite Material Co., Ltd., Ningbo, China). The PEEK pellets were dried at 110 °C for 6 h in a vacuum oven. The specimens were prepared by injection molding with a mold temperature of 190 °C, a nozzle temperature of 385 °C, a heating zone temperature of 380 °C, a screw rotation speed of 50%, and a back pressure of 3 MPa. After injection, the specimens were removed and allowed to cool naturally to room temperature, and then trimmed to remove flash. The quasi-static tensile specimens measured 170 mm × 20 mm × 4 mm. The gauge length of the quasi-static specimen was 80 mm. According to the GB/T1040 [38], a dog bone-shaped test piece was designed. The test specimens were prepared by preheating compression molding, and the geometric shape, dimensions and standard test piece are shown in Figure 2.

The quasi-static uniaxial tensile test was conducted using the LE5205 microcomputer-controlled electronic universal testing machine (Shanghai Lishi Scientific Instrument Corporation, Shanghai, China). The hydraulic grips provide sufficient clamping force during loading to avoid specimen slippage. The testing machine is equipped with a microelectronic controlled high-temperature furnace, which can adjust the internal temperature through a heating devices or injecting liquid nitrogen. To ensure uniform surface temperature distribution on the specimen, a constant heating rate was set, and the clamped specimen was maintained in the high-temperature furnace for at least 15 min after reaching the target temperature according to Standard GB/T9979 [39]. During heating, the crosshead was allowed to accommodate thermal expansion so that the specimen remained stress-free. After the temperature stabilized and the load signal became stable, the crosshead position was adjusted to bring the load to zero, and this position was taken as the initial reference for strain measurement. The test was then started immediately.

The quasi-static uniaxial tensile tests were conducted at five temperatures (20 °C, 50 °C, 80 °C, 110 °C, 140 °C), with four strain rates (10^−4^ s^−1^, 10^−3^ s^−1^, 10^−2^ s^−1^, 10^−1^ s^−1^) applied for each temperature. For each test condition (temperature and strain rate), three replicate specimens were tested to ensure experimental reliability and validity. Due to the large dataset and satisfactory repeatability, the stress–strain curve closest to the mean value was selected as the representative result for each condition. Prior to testing, all specimens were dimensionally measured using vernier calipers, and the recorded dimensions were subsequently used for data processing. The strain rate was controlled by setting the crossbeam displacement speed of the testing machine. Calculations confirmed that this method could maintain a constant engineering strain rate. The gauge length of the specimen was 80 mm, as shown in Figure 2a. The engineering strain rate was calculated as the crosshead speed divided by the gauge length. The four strain rates of 10^−4^ s^−1^, 10^−3^ s^−1^, 10^−2^ s^−1^ and 10^−1^ s^−1^ corresponded to crossbeam speeds of 0.48 mm/min, 4.8 mm/min, 48 mm/min, 480 mm/min, respectively. During the testing process, the load and displacement data were provided by the sensor of the test machine.

Assuming constant volume during deformation, the true stress *σ_T_* and true strain *ε_T_* can be expressed as [40]:

*σ_T_* = *σ_E_*(1 + *ε_E_*) (1)

(2) $$\varepsilon_{T}=\ln(1+\varepsilon_{E})$$

We have explicitly stated that the engineering stress–strain data were converted to true stress–true strain using Equations (1) and (2), and that all subsequent analyses use true quantities.

### 2.2. Tensile Mechanical Responses and Analysis

**Figure 3:**
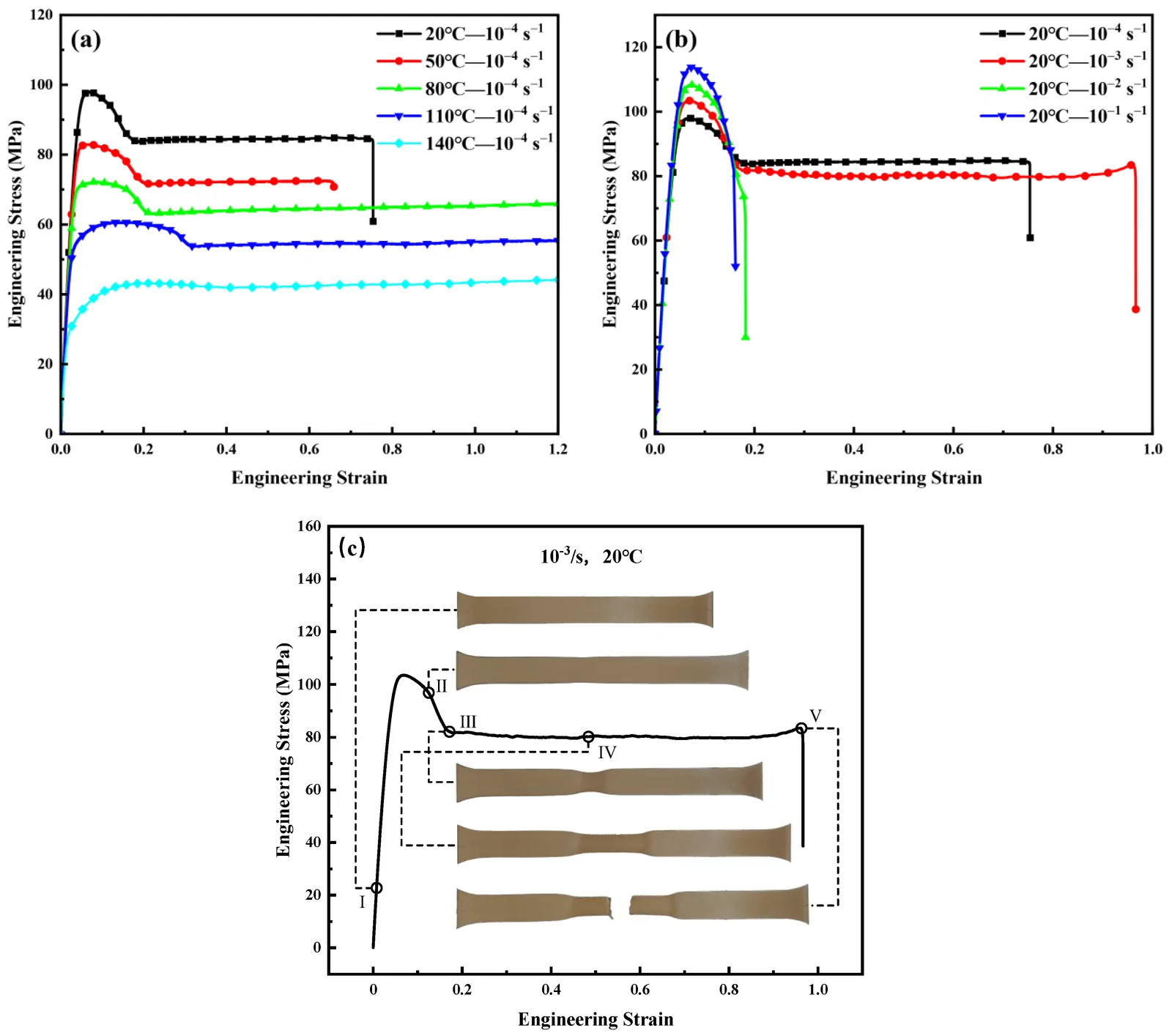
Representative engineering stress–strain curves of PEEK in quasi-static tensile tests. (**a**) 10^−4^ s^−1^, (**b**) 20 °C, (**c**) 10^−3^ s^−1^ and 20 °C.

Uniaxial tensile tests of PEEK material were performed, and obtained multiple sets of load–displacement data. A representative engineering stress–strain curve is plotted as shown in Figure 3a,b. And a schematic diagram of the strain response of PEEK material at 10^−3^ s^−1^ strain rates and 20 °C is provided in Figure 3c.

As shown in Figure 3a,b, the quasi-static uniaxial tensile response of PEEK material exhibits typical nonlinear viscoelastic characteristics at different temperatures and strain rates, and has pronounced temperature and strain rate dependence. The uniaxial tensile response of PEEK material can be divided into three stages: (1) Pre-yield stage. Region I in Figure 3c represents short-range elastic deformation. The stress increases until reaching the yield stress with strain. (2) Yield stage. Strain softening [41] starts at the yield stress (local maximum stress). The yield point can be clearly observed immediately before the onset of necking. Region II in Figure 3c can observe that necking begins to appear. (3) Post-yield stage. Regions III and IV in Figure 3c depict the necking process in areas with relatively weak resistance to deformation, and a nearly constant flow stress is observed [25,42]. And region V represents the final fracture of the PEEK test piece.

In general, the quasi-static uniaxial tensile response of PEEK material exhibits significant dependence on temperature and strain rate, which represents characteristic features associated with viscoelastic mechanics. Due to the disruption of the uniaxial stress state of PEEK specimens during necking, this study will focus primarily on the viscoelastic mechanical behavior of PEEK before yielding.

**Figure 4:**
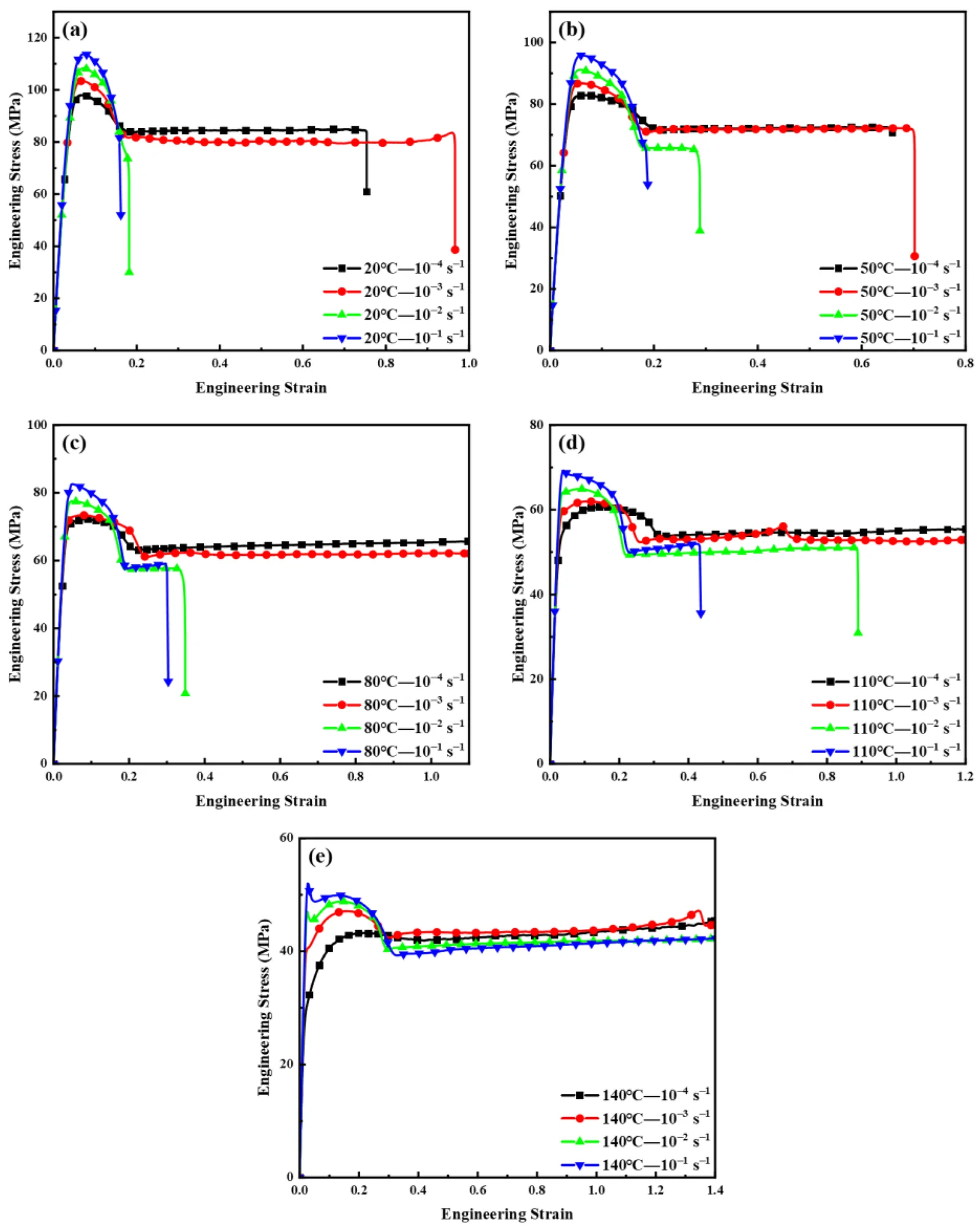
Comparison of engineering stress–strain curves of PEEK. (**a**) 20 °C, (**b**) 50 °C, (**c**) 80 °C, (**d**) 110 °C, (**e**) 140 °C.

Figure 4 shows the engineering stress–strain curves obtained from quasi-static uniaxial tensile tests conducted at different strain rates under five temperatures. It can be seen that the tensile response of PEEK material has pronounced strain rate sensitivity. It can be observed that the yield stress of PEEK material exhibits a positive correlation with strain rate.

**Figure 5:**
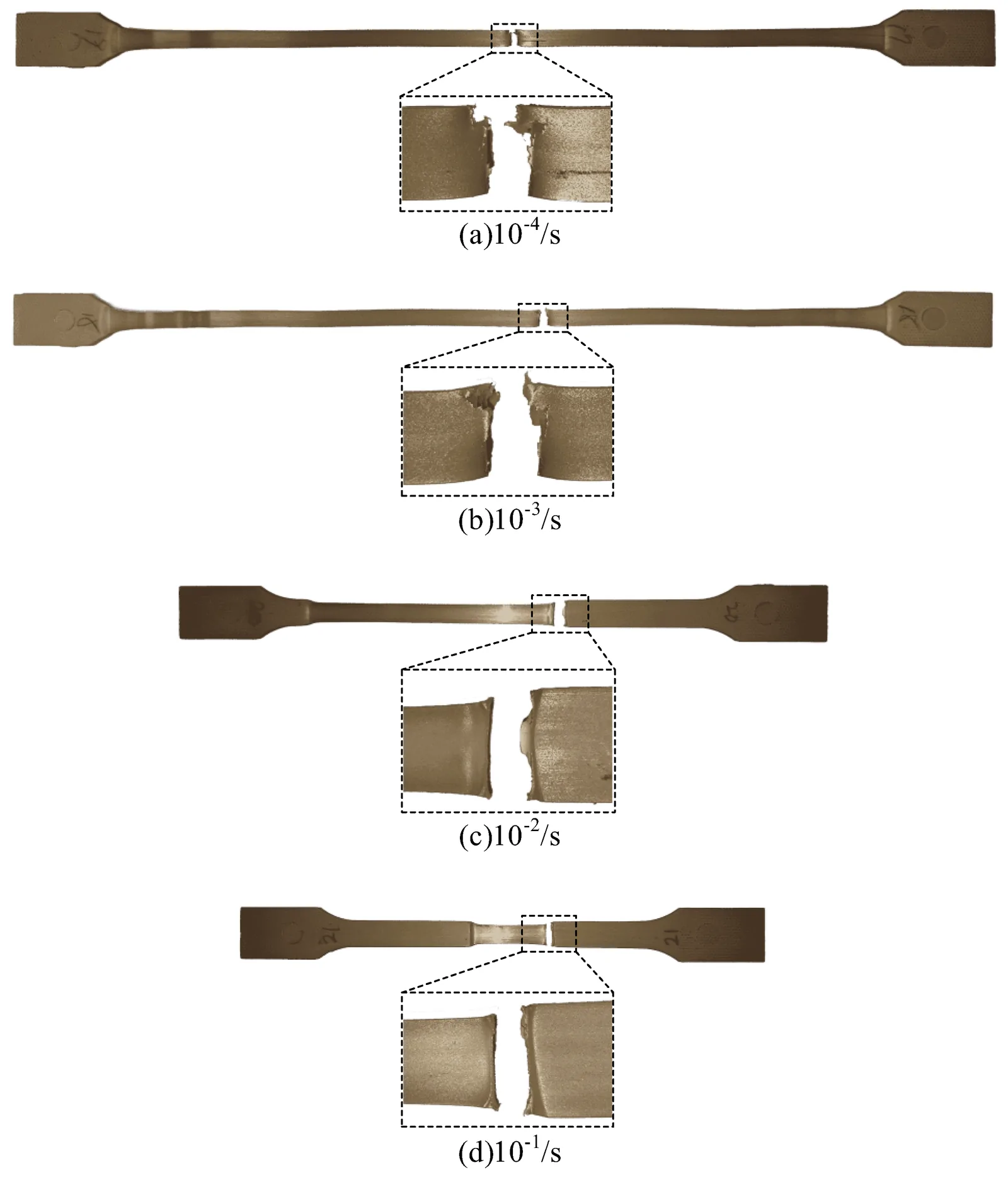
Comparison of fracture shapes of PEEK specimens at different strain rates at 110 °C (**a**) 10^−4^ s^−1^, (**b**) 10^−3^ s^−1^, (**c**) 10^−2^ s^−1^, (**d**) 10^−1^ s^−1^.

The yield stress increases with elevated strain rate, while the elastic modulus also slowly increase. Strain rate also has a significant impact on the failure mode of materials. Figure 5 shows a comparison of the fracture surface shapes of PEEK material after quasi-static uniaxial tensile tests under 110 °C. Under higher strain rates (10^−1^ s^−1^, 10^−2^ s^−1^), the fracture cross-section of the test piece is relative smooth and flat, accompanied by serrated features. This morphology is attributed to the rapid loading process, wherein the material’s internal structure could not accommodate the abrupt stress variations. The inherent defects and microcracks propagated rapidly under the applied stress, ultimately leading to catastrophic failure. The relatively smooth and flat nature of the fracture surface indicates that both sides of the fracture experienced essentially identical stress states during propagation, with no significant relative displacement or shear deformation occurring across the fracture plane. And under low strain rates (10^−3^ s^−1^, 10^−4^ s^−1^), the cross-section of the fracture surface of the test piece has tearing traces, and the fracture surface shape is rough and irregular. The failure mode exhibits more obvious ductile fracture. This phenomenon is attributed to the combined effects of internal plastic deformation of the material and various energy dissipation processes, which collectively decelerate crack propagation through the material. The resultant significant relative displacement and shear deformation across the fracture plane lead to the formation of an irregular fracture surface morphology. This further highlights the high tensile strength, excellent toughness and ductility of PEEK material under low strain rate conditions.

In the pre-yield stage, the yield strength increases with rising strain rate, exhibiting a distinct strain-rate strengthening effect. In the post-yield stage, although the failure mode remains ductile fracture across all strain rates, a more pronounced brittle characteristic emerges at higher strain rates. Conversely, at lower strain rates, the material demonstrates enhanced toughness and ductility.

**Figure 6:**
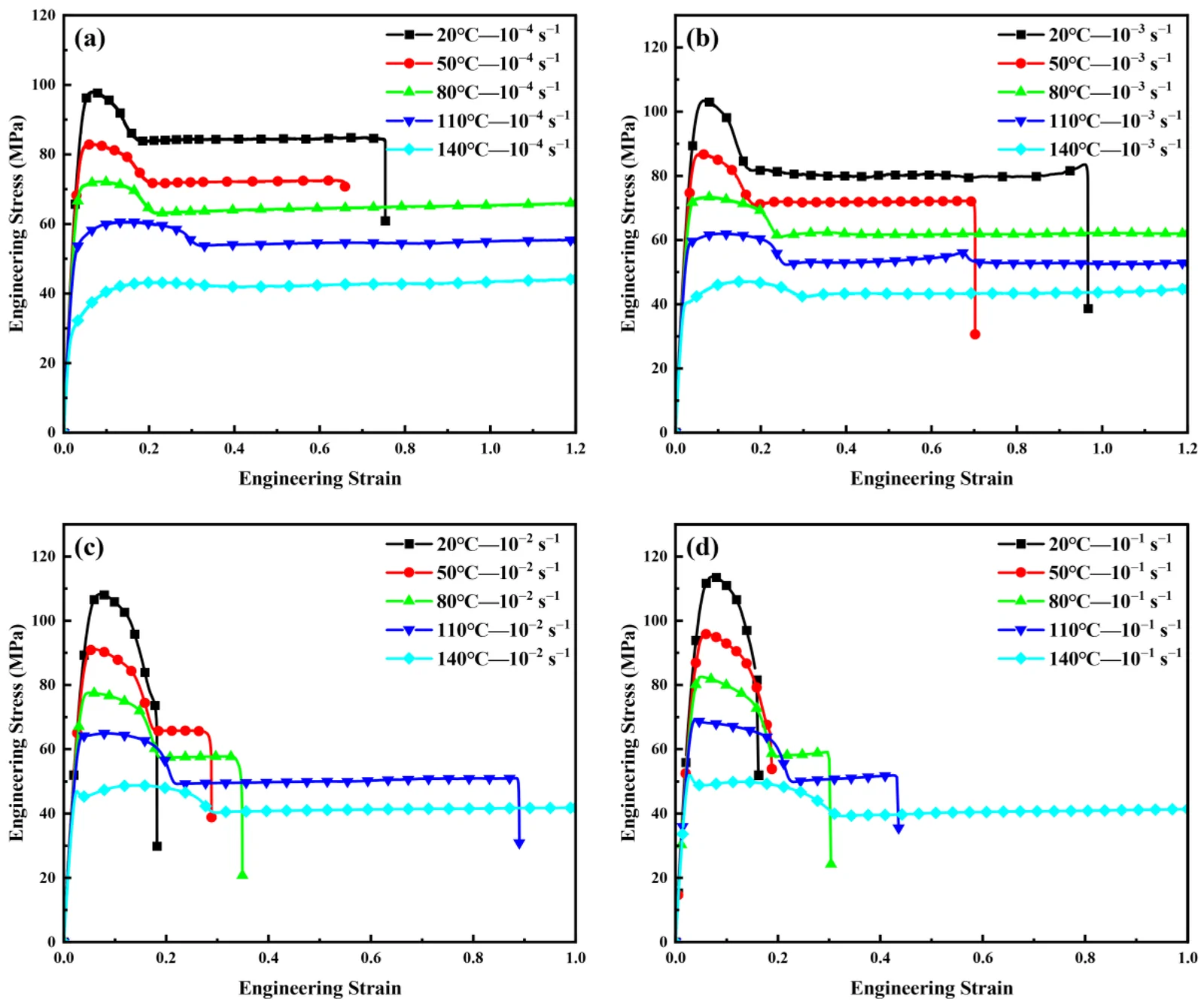
Comparison of engineering stress–strain curves of PEEK. (**a**) 10^−4^ s^−1^, (**b**) 10^−3^ s^−1^, (**c**) 10^−2^ s^−1^, (**d**) 10^−1^ s^−1^.

**Figure 7:**
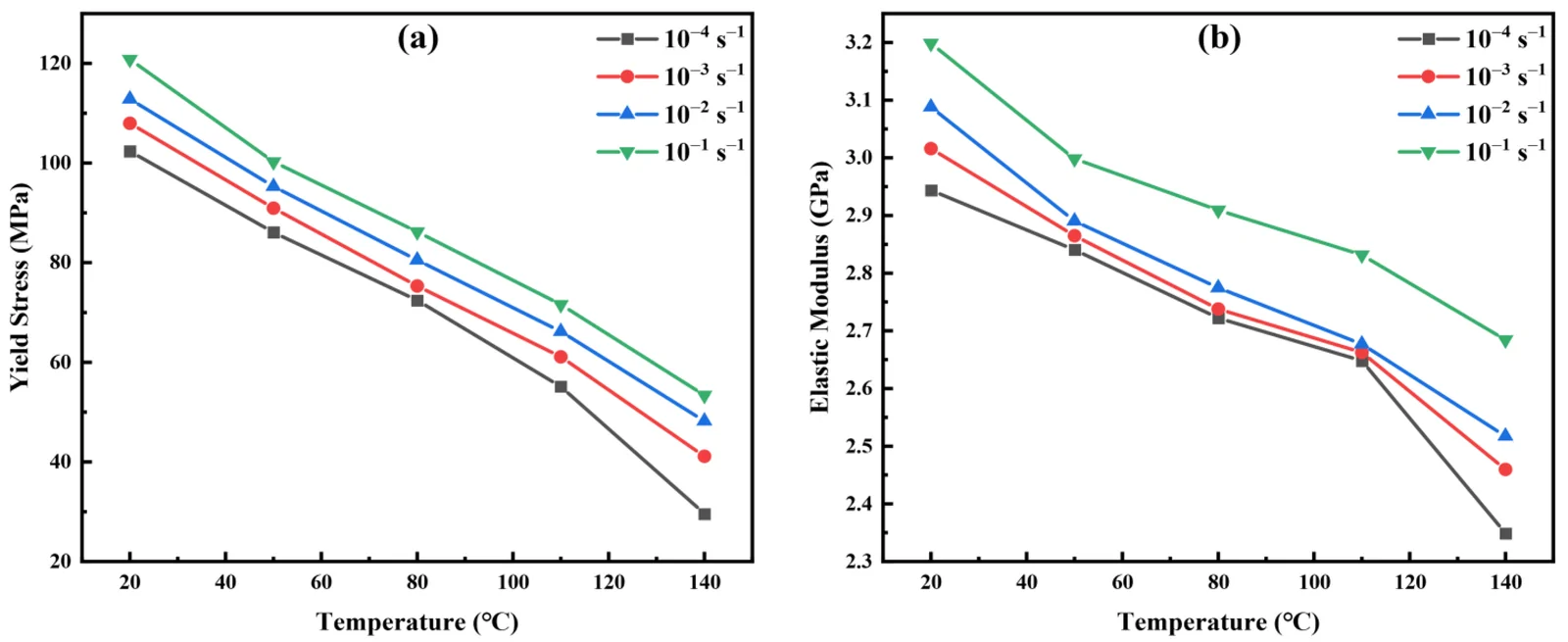
Characterization of mechanical properties of PEEK at the same strain rate. (**a**) Yield strength. (**b**) Initial elastic modulus.

Temperature is also one of a critical factor influencing on the tensile response of PEEK material. Figure 6 shows a comparison of the tensile engineering stress–strain curves of PEEK material at different temperatures. PEEK exhibits a pronounced nonlinear mechanical response prior to yielding and its yielding behavior demonstrates significant temperature dependence. Figure 7 shows that with increasing temperature, the yield stress decreases nearly linearly, while the initial elastic modulus progressively diminishes. The stress drop rate following the yield point reflects the plastic deformation capacity of the material and is one of the critical indicators for judging the enhancement or weakening of material toughness. As shown in Figure 6, the toughness of PEEK increases progressively with rising temperature. This is manifested as a deceleration in the rate of stress decrease with increasing strain after yield. From a microstructural perspective, enhanced toughness in semi-crystalline polymers is typically associated with lamellar slip, lamellar fragmentation, and the orientation of amorphous chains. These microstructural rearrangements enable greater absorption of strain energy, providing additional energy dissipation during stress relaxation and consequently promoting more extensive deformation and improved ductility. Conversely, reduced toughness is characterized by an accelerated stress decrease rate with increasing strain. This phenomenon arises from microstructural changes such as lamellar fragmentation, microvoid formation, and localized shear deformation which induce stress concentration and promote localized fracture.

**Figure 8:**
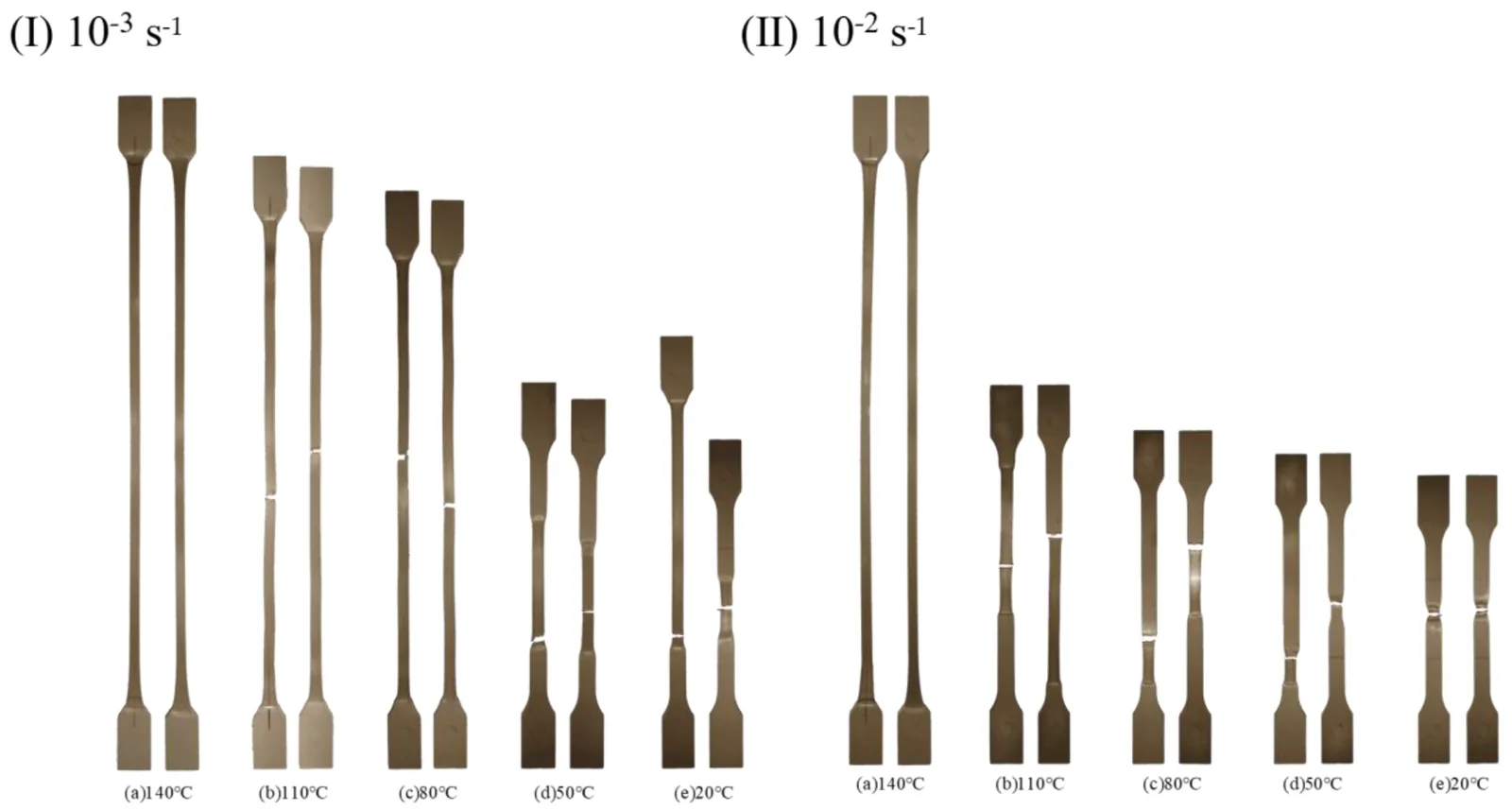
Comparison of test piece lengths after different temperature tests of PEEK at (**I**) 10^−3^ s^−1^, (**II**) 10^−2^ s^−1^: (**a**) 140 °C, (**b**) 110 °C, (**c**) 80 °C, (**d**) 50 °C, (**e**) 20 °C.

Elongation at break is also a critical indicator to describe the plastic deformation capacity of materials, and can be used to evaluate the toughness and ductility. As shown in Figure 8, the elongation at break of the material is often higher at elevated temperatures. But at lower temperatures, it exhibits different situations at different strain rates. As low strain rates, the material has sufficient time to undergo plastic deformation during the tensile process, so the material requires higher stress to induce failure. In this case, the failure strain is usually high but exhibits significant variability and instability. Conversely, at high strain rates, the material is subjected to higher stress over a short period of time, promoting easier plastic deformation and failure. And the failure strain is generally lower and more stable, exhibiting reduced variability. As shown in Figure 8, the phenomenon that the elongation at break of the material increases with rising temperature is more readily observed at a strain rate of 10^−2^ s^−1^.

### 2.3. High-Temperature Rheological Behavior

**Figure 9:**
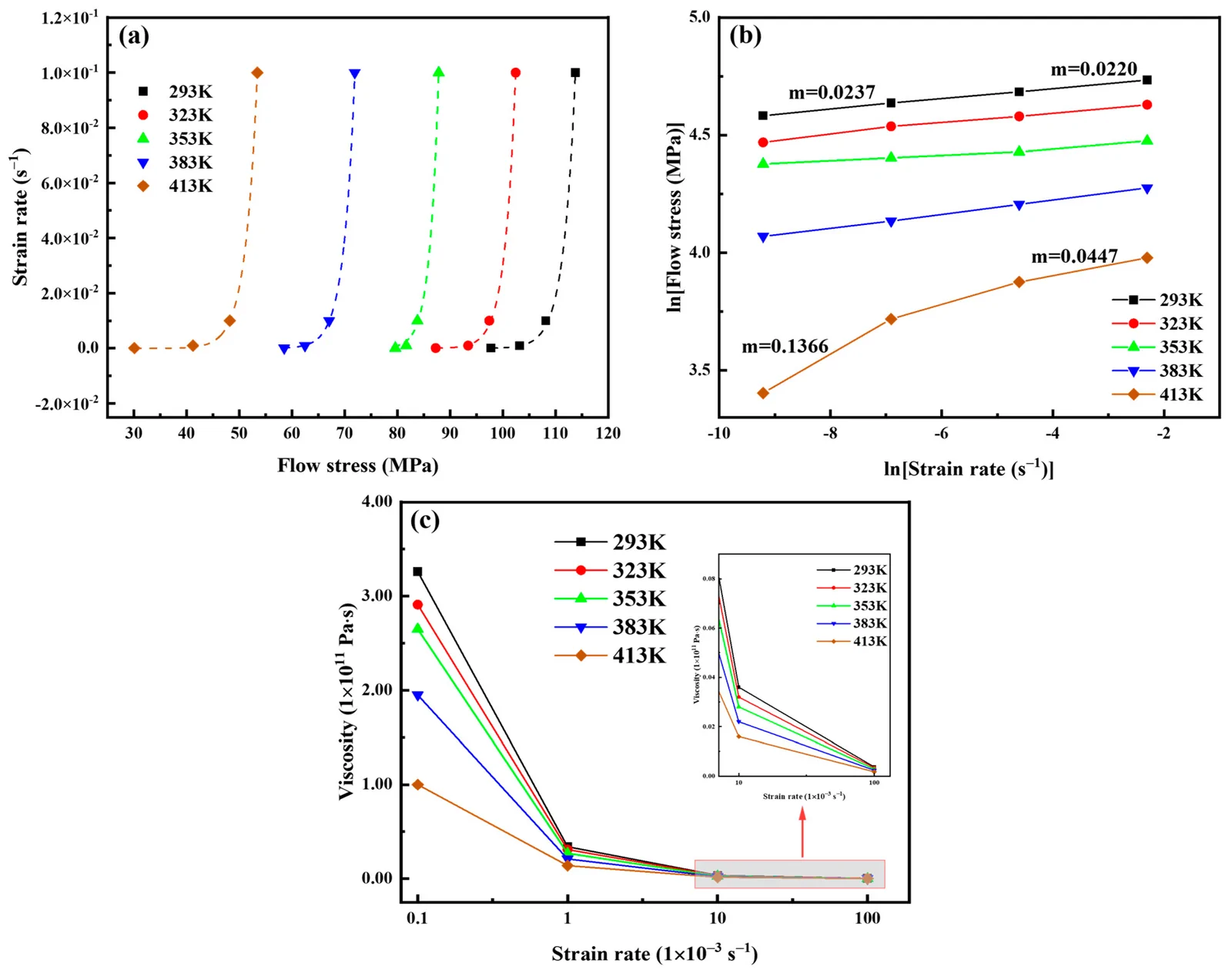
(**a**) Variation in the steady-flow stress with the applied strain rate. (**b**) Variation in the steady-state flow with strain rate of PEEK at different tensile temperatures. (**c**) The viscosity dependence profile on temperature and strain rate.

Based on the experimental results presented above, it is demonstrated that the plastic deformation of PEEK similarly exhibits temperature and strain rate sensitivity. This need to systematically investigate the rheological behavior coupled with quantitative assessment and comparative analysis of the strain rate sensitivity of PEEK. In the case of homogenous deformation and high temperature, it is generally accepted that a transition from a Newtonian to non-Newtonian viscous flow occurs and this transition shows a strong dependence on temperature and strain rate [43]. The variation in the steady-state flow with the strain rate, the strain rate sensitivity index and the viscosity at different temperatures and strain rate of PEEK is given in Figure 9.

Strain rate at various temperatures. The flow stress exhibits temperature and strain rate sensitivities. With the strain rate rising, the flow stress will increase at the same temperature. And with the rate of strain rising, this trend becomes more pronounced. Temperature decline causes an increase in steady-state flow stress at the same strain rate.

According to the free volume theory, the strain rate at high temperatures under uniaxial tensile tests and takes the form of [44]:

(3) $$\dot{\varepsilon }=2c_{f}v_{D}\exp\left(-\frac{\Delta G^{m}}{kT}\right)\sinh\left(\frac{\sigma V}{2\sqrt{3}kT}\right)={\dot{\varepsilon }}_{0}\sinh\left(\frac{\sigma V}{2\sqrt{3}kT}\right)$$

where Δ*G^m^* is the activation energy, *V* is the activation volume. *k* = 1.38 × 10^−23^*J*/*K* is the Boltzmann constant, and ${\dot{\varepsilon }}_{0}$ is a rate factor.

Equation (3) can be used to describe the mechanical properties of PEEK at high temperature where the deformation is homogenous. And activation volume *V* and frequency factor ${\dot{\varepsilon }}_{0}$ could be obtained from fitting the experimental data of steady-state flow stress and strain rate of PEEK materials at different temperatures.

Viscosity is a critical parameter in studying the plastic deformation of PEEK. Apparent viscosity can be rigorously derived from the relationship of stresses and strains:

(4) $$\eta =\frac{\tau_{s}}{{\dot{\gamma }}_{s}}=\frac{\sigma }{3\dot{\varepsilon }}$$

where *σ* and $\dot{\varepsilon }$ are the stress and strain from uniaxial tensile tests [45]. ${\dot{\gamma }}_{s}=\sqrt{3}\times \dot{\varepsilon }$ is the equivalent shear strain, and $\tau_{s}=\frac{\sigma }{\sqrt{3}}$ is the equivalent shear stress.

As shown in Figure 9c, under lower temperature conditions, plastic deformation manifests heterogeneity with viscosity exhibiting dependence on strain rate. Conversely, at elevated temperatures, plastic deformation transitions toward homogeneity while viscosity demonstrates diminished sensitivity to strain rate variations. The apparent viscosity decreases with increasing temperature, reflecting enhanced thermally activated chain mobility. Under such thermal conditions, the strain rate dependence of viscosity becomes diminished, demonstrating reduced strain rate sensitivity. Strain rate sensitivity is a primary parameter characterizing the rheological behavior and the fundamental physical mechanisms underlying deformation, which is defined as

(5) $$m=\frac{\partial \log\sigma_{\mathrm{flow}}}{\partial \log\dot{\varepsilon }}$$

As the parameter *m* approaches unity, the plastic deformation process is increasingly close to the Newtonian viscous flow. As shown in Figure 9b, the strain rate sensitivity *m* declines from 0.0237 to 0.0220 remaining nearly constant at low temperature at 293 K, which reflects a typical non-Newtonian behavior. In contrast, when the tensile temperature increases to 413 K (close to *T_g_*), *m* decreases from 0.1366 to 0.0447. Although this value is still far below 1.0 and the material remains highly non-Newtonian, the decrease in *m* indicates a weakening of strain rate sensitivity and a trend toward Newtonian viscous flow.

## 3. Constitutive Model of PEEK

### 3.1. Constitutive Model

Linear viscoelasticity can be described by simplified mechanical models composed of linear springs and Newtonian dashpots as fundamental elements. Within the linear viscoelastic regime, the linear spring represents elastic deformation, while the Newtonian dashpot characterizes viscous flow. According to the Boltzmann superposition principle, these two elements can act independently or be superposed. For ideal linear springs and Newtonian dashpots, they obey Hooke’s law and Newton’s law of viscosity, respectively, and their constitutive relations can be expressed as

*σ* = *Eε* (6)

(7) $$\sigma =\eta \dot{\varepsilon }$$

Equation (6) represents the elastic response, while Equation (7) represents the viscous response. Here, *E* is the elastic coefficient of the linear spring, *η* is the viscosity coefficient of the Newtonian dashpot, and *σ*, *ε*, and $\dot{\varepsilon }$ denote the stress, strain, and strain rate under small deformation conditions, respectively. By combining linear springs and Newtonian dashpots in different configurations, various mechanical models can be established, such as the Maxwell model, Kelvin–Voigt model, standard linear solid model, and Burgers model [46].

(1) Two-Element Model: Maxwell Model

**Figure 10:**
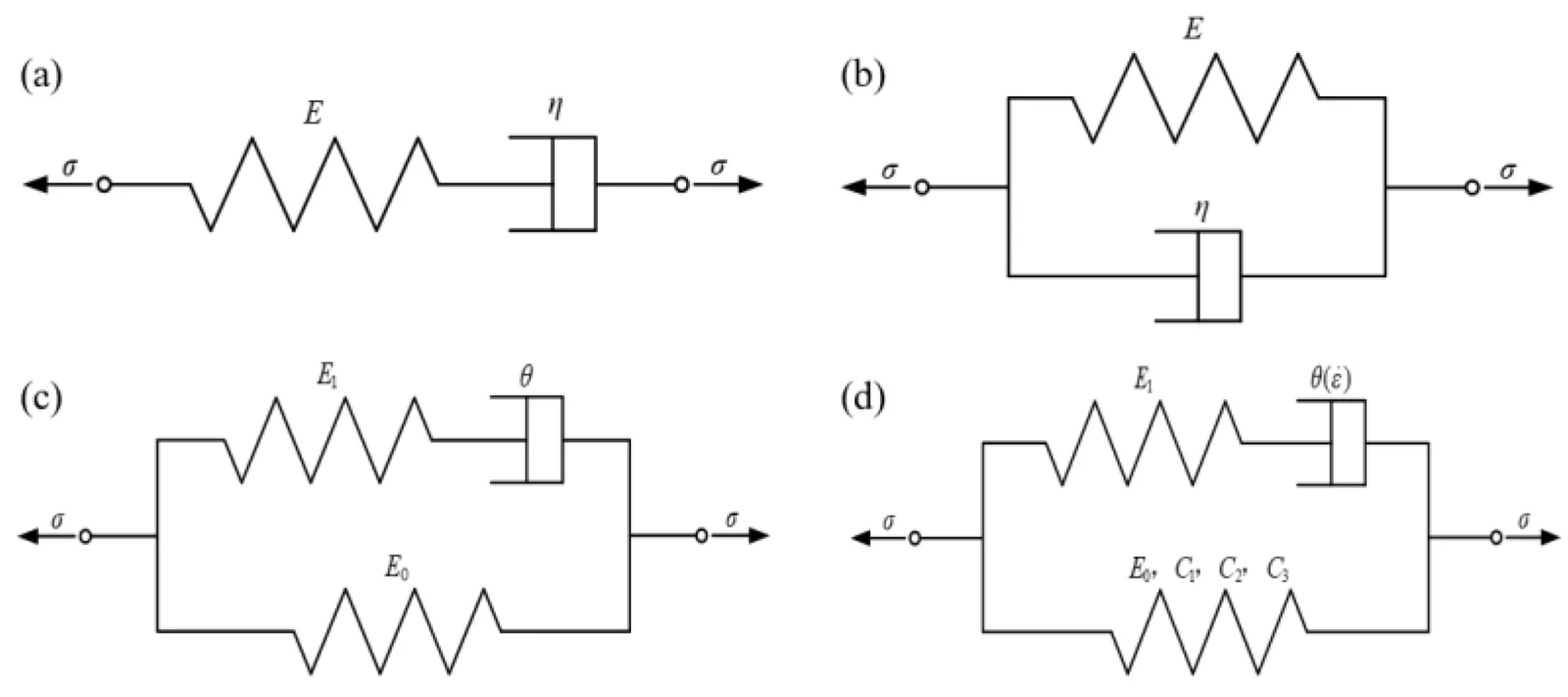
(**a**) Maxwell Model, (**b**) Kelvin Model, (**c**) three-parameter model, (**d**) seven parameters nonlinear viscoelastic constitutive model.

The simplest series combination in linear viscoelastic mechanical modeling is the Maxwell model, which consists of a linear spring and a Newtonian dashpot connected in series, as illustrated in Figure 10a. Since the stresses acting on both elements are equal, the total strain is the sum of the strains in the individual elements, which can be expressed as

(8) $$\varepsilon =\varepsilon_{e}+\int_{0}^{t}\dot{\varepsilon }dt$$

where *ε_e_* = *σ*/*E* represents the strain of the linear spring, and $\dot{\varepsilon }=\sigma /\eta$ denotes the strain rate of the Newtonian dashpot, the time integral of which gives the strain of the dashpot. Differentiating Equation (8) with respect to time yields the differential form of the constitutive equation for the Maxwell model:

(9) $$\dot{\varepsilon }=\frac{\dot{\sigma }}{E}+\frac{\sigma }{\eta }$$

By applying a constant strain *ε* = *ε*_0_, the Maxwell model is capable of describing stress relaxation behavior. Under this condition, $\dot{\varepsilon }=0$, and thus the following expression can be obtained:

(10) $$\dot{\sigma }=-\frac{\sigma }{\eta }E$$

At *t* = 0, the initial stress is *σ* = *Eε*_0_. Solving the differential equation yields

(11) $$\sigma =E\varepsilon_{0}e^{-\frac{t}{\tau }}$$

where *τ* = *η*/*E* represents the relaxation time. Equation (8) indicates that, at *t* = 0, the material exhibits an instantaneous stress response, after which the stress *σ* decays exponentially with the relaxation time *τ*.

Due to the presence of the Newtonian dashpot in the Maxwell model, the material cannot maintain a finite deformation under constant stress but instead undergoes continuous flow, thereby exhibiting behavior intermediate between that of a fluid and a solid. Furthermore, the relaxed stress asymptotically decays to zero after an infinitely long period. In real materials, however, stress relaxation is a progressive process in which the stress does not completely vanish over infinite time. Evidently, the Maxwell model does not provide an accurate description of the stress relaxation behavior of viscoelastic materials.

(2) Two-Element Model: Kelvin Model

As shown in Figure 10b, the Kelvin model consists of a linear spring and a Newtonian dashpot connected in parallel.

In this configuration, the strains in both elements are identical, and the total stress is the sum of the stresses carried by each element. The fundamental stress–strain relationship can be expressed as follows:

(12) $$\sigma =E\varepsilon +\eta \dot{\varepsilon }$$

where *Eε* represents the stress in the linear spring, and $\eta \dot{\varepsilon }$ denotes the stress in the Newtonian dashpot.

Applying a constant strain *ε* = *ε*_0_ yields $\dot{\varepsilon }=0$. Substituting this condition into Equation (12) gives

*σ* = *Eε*_0_ (13)

As can be seen from Equation (13), under a constant applied strain *ε* = *ε*_0_, the stress sustained by the model is a constant value *Eε*_0_. Since a Newtonian dashpot is connected in parallel with the spring, an infinite stress would be required to produce an instantaneous strain in the model, which contradicts reality. Therefore, it is evident that the Kelvin model cannot describe stress relaxation behavior.

From the above analysis, it can be seen that the structural configurations of the Maxwell and Kelvin models are overly simplistic and cannot adequately describe the mechanical behavior of viscoelastic materials. Consequently, more complex models are required to accurately capture the viscoelastic response of such materials.

As discussed above, by combining linear springs and Newtonian dashpots in various configurations, constitutive models suitable for different materials can be established. Figure 10c shows a three-parameter model formed by connecting a linear spring in series with a linear Maxwell unit, which has been widely employed in investigating the viscoelastic behavior of numerous materials. The constitutive equation of this model is given by

(14) $$\sigma =E_{0}\varepsilon +E_{1}\int_{0}^{t}\dot{\varepsilon }(\tau )e^{-\frac{t-\tau }{\theta }}d\tau$$

where *E*_0_ and *E*_1_ are the elastic coefficients of the linear springs, *θ* is the relaxation time of the linear Maxwell unit, $\dot{\varepsilon }$ is the applied strain rate, and *τ* is an integration variable. This model is capable of describing the stress relaxation and creep behavior of materials; however, it is only valid for capturing viscoelastic responses within a low strain rate range and is not applicable to the description of nonlinear viscoelasticity.

A phenomenological nonlinear viscoelastic constitutive model considering both strain rate sensitivity and temperature dependence is proposed. The model is a seven parameters model consisting of a parallel combination of a fourth-order nonlinear spring unit and a nonlinear Maxwell unit, as shown in Figure 10d. The relationship between the constitutive equation of the model is as follows—the stress *σ* and strain *ε* in the constitutive equations denote true stress and true strain:

(15) $$\begin{array}{l} \sigma =E_{0}\varepsilon +C_{1}\varepsilon^{2}+C_{2}\varepsilon^{3}+C_{3}\varepsilon^{4}+E_{1}\int_{0}^{t}\dot{\varepsilon }(\tau )e^{-\frac{t-\tau }{\theta }}d\tau ,\\ \theta =\theta_{0}{\left(\frac{\dot{\varepsilon }}{{\dot{\varepsilon }}_{0}}\right)}^{-\beta } \end{array}$$

where *E*_0_, *C*_1_, *C*_2_ and *C*_3_ are the elastic coefficients of the nonlinear spring, *E*_1_ and *θ* are the elastic constant and relaxation times of nonlinear Maxwell element, respectively. And the relaxation time *θ* is defined as a function of strain rate $\dot{\varepsilon }$, $\dot{\varepsilon }$ is the strain rate, *θ*_0_ and *β* are undetermined parameters. ${\dot{\varepsilon }}_{0}$ is the reference strain rate, and in this paper, ${\dot{\varepsilon }}_{0}=1.0/s$.

The true data before yielding at the same temperatures but different strain rates were fitted by using the least squares method. Combined with Equation (15) parameter *θ* was determined. The values of the model parameters are shown in Table 1. It is evident that the six parameters of the constitutive model, excluding *β*, exhibit significant variations with temperature. To describe the temperature-dependent stress–strain relationship of PEEK, these six parameters are treated as functions of temperature, and the constitutive Equation (16) is modified as follows:

(16) $$\sigma =E_{0}(T)\varepsilon +C_{1}(T)\varepsilon^{2}+C_{2}(T)\varepsilon^{3}+C_{3}(T)\varepsilon^{4}+E_{1}(T)\int_{0}^{t}\dot{\varepsilon }(\tau )e^{-\frac{t-\tau }{\theta_{0}(T){\left(\frac{\dot{\varepsilon }}{{\dot{\varepsilon }}_{0}}\right)}^{-\beta }}}d\tau$$

where *T* is temperature, and *E*_0_(*T*), *C*_1_(*T*), *C*_2_(*T*), *C*_3_(*T*), *E*_1_(*T*) and *θ*_0_(*T*)are six functions parameters of temperature. The six function parameters were obtained through polynomial interpolation. The dimensionless expressions of the interpolation functions describing the relationship between model parameters and temperature and determination coefficients *R*^2^(*cod*) are shown in Table 2. To determine the termination point of the stress–strain curve in the nonlinear viscoelastic constitutive model, the yield strain is fitted as a linear function of temperature. The expression of the interpolation function is as follows:

(17) $$\varepsilon_{\mathrm{yield}}=0.06511-0.00297467T$$

where *ε_yield_* represents the yield strain.

It should be noted that the proposed constitutive model is restricted to the pre-yield stage. It does not apply to post-yield softening, necking, or large plastic deformation, and its use should therefore be limited to small-strain, pre-yield conditions.

### 3.2. Prediction and Validation

**Figure 11:**
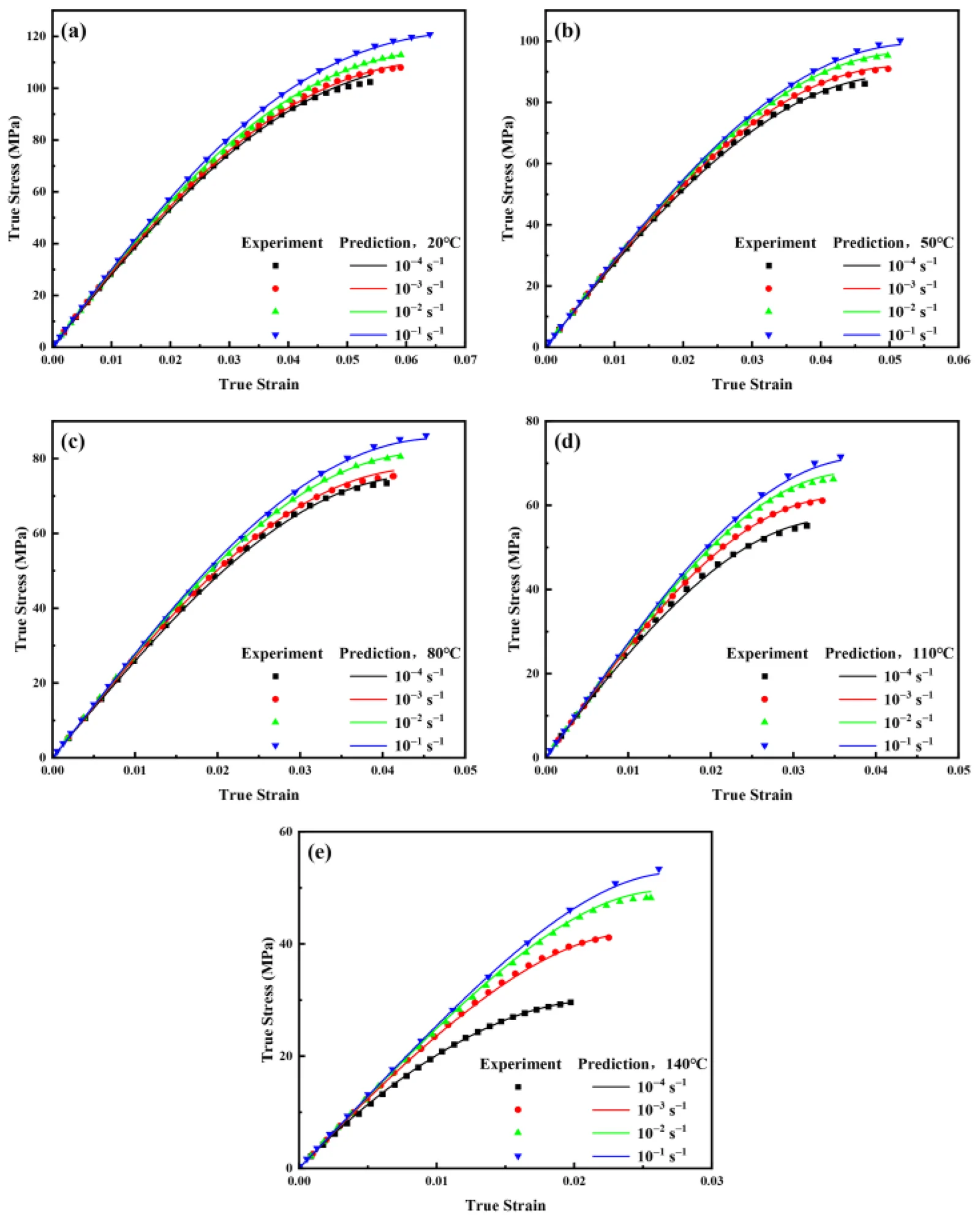
The true stress–strain curve of PEEK at same temperature before yielding and model prediction. (**a**) 20 °C, (**b**) 50 °C, (**c**) 80 °C, (**d**) 110 °C, (**e**) 140 °C.

**Figure 12:**
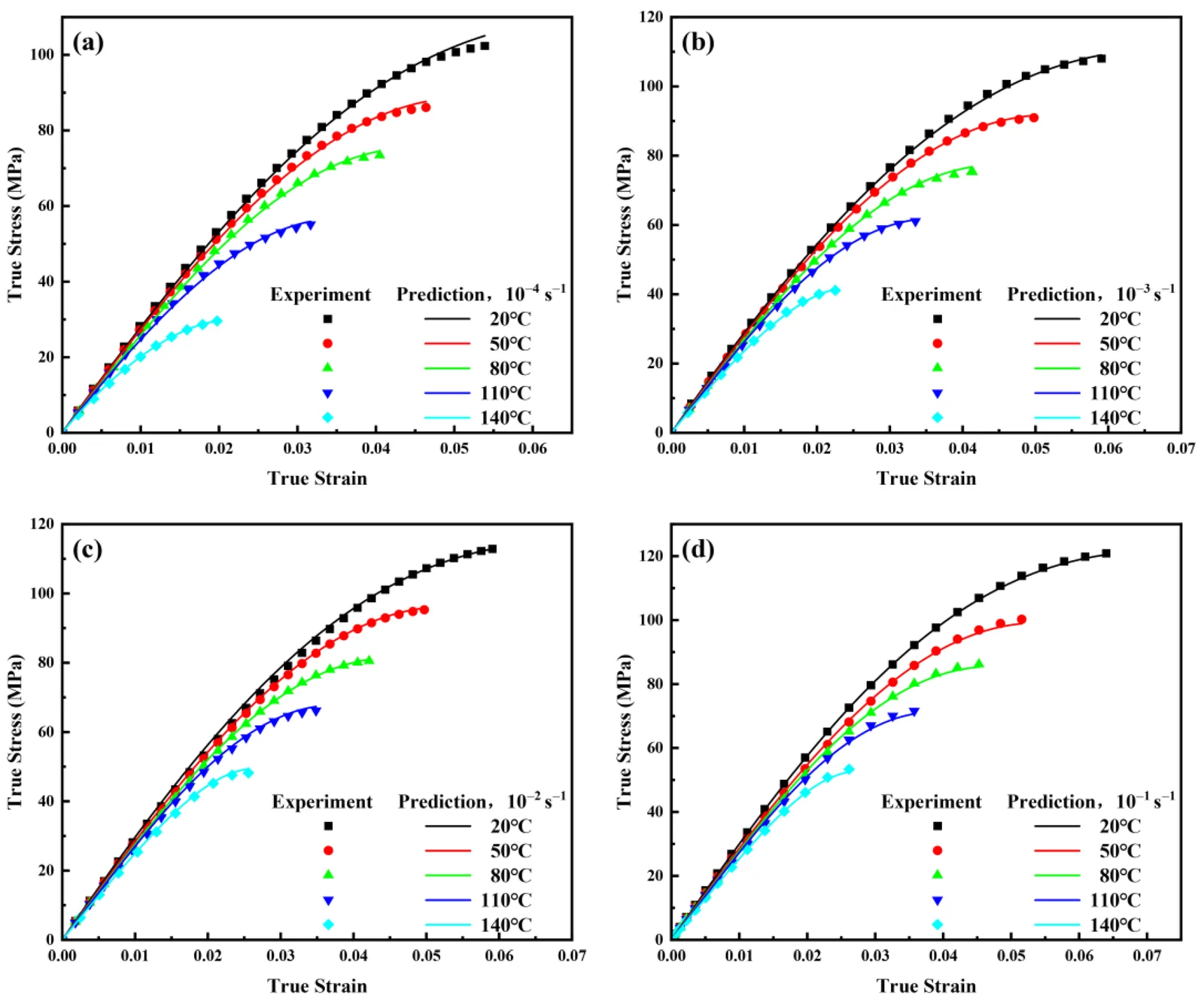
The true stress–strain curve of PEEK at same strain rate before yielding and model prediction. (**a**) 10^−4^ s^−1^, (**b**) 10^−3^ s^−1^, (**c**) 10^−2^ s^−1^, (**d**) 10^−1^ s^−1^.

Figure 11 and Figure 12, respectively, show a comparison between experimental data and model predictions for the true stress–strain curves of PEEK before yielding given under the same temperature and strain rate conditions. The model provides accurate predictions of the experimental results under most conditions, except for a slight deviation (less than 3%) at temperature of 20 °C and 50 °C with a strain rate of 10^−4^ s^−1^. It is noteworthy that the experimental data obtained at 50 °C were not used to determine the parameters of the constitutive model. This demonstrates the correctness and reliability of the constitutive model used in characterizing and predicting the quasi-static nonlinear viscoelastic mechanical behavior of PEEK over a wide temperature range.

## 4. Conclusions

Through the following experiments and analysis, this paper obtained the influence of temperature and strain rate on the performance of PEEK. And a seven-parameter modified constitutive model was derived from physical and mechanical characterization, respectively, providing a data basis for the application of PEEK matrix and future carbon fiber-reinforced PEEK composites. This model is specifically tailored to accurately characterize the mechanical response of PEEK within this narrowly defined, but practically significant, window of strain rates and temperatures, thereby providing a more precise and physically interpretable tool for applications in this regime.

Quasi-static uniaxial tensile tests on PEEK material within a temperature range of 20 °C to 140 °C and a strain rate range of 10^−4^ s^−1^ to 10^−1^ s^−1^ were conducted. The experimental results demonstrate that the tensile mechanical response of PEEK exhibits significant temperature and strain rate dependence. With increasing temperature, the yield strength of PEEK decreases nearly linearly, while the elastic modulus gradually diminishes. Concurrently, its toughness and ductility improve, and brittleness is reduced. With increasing strain rate, both the yield strength and modulus of PEEK increase.

Experimental evidence demonstrates that PEEK materials exhibit a transition trend from non-Newtonian flow to Newtonian flow blew temperatures Tg. With the strain rate rising, the steady-flow stress will increase at the same temperature. The flow stress also exhibits temperature sensitivity. With temperature declining, the steady-state flow stress at the same strain rate increase. The strain rate sensitivity index increases and the viscosity decrease with temperature rising.

A seven-parameter modified constitutive model was proposed to characterize the tensile mechanical response of PEEK before yielding under varying temperature and strain rate conditions. The results demonstrate that the constitutive model can accurately predict the nonlinear viscoelastic response of PEEK prior to yielding in quasi-static uniaxial tensile tests within the strain rate and temperature ranges investigated in this paper.

### Limitations

This study has several limitations that should be acknowledged. First, the investigated strain rate range is restricted to 10^−4^–10^−1^ s^−1^, which covers only quasi-static loading conditions. Second, the test temperatures (20–140 °C) are all below and relatively close to the glass transition temperature of PEEK (*T_g_* ≈ 143 °C). Therefore, the proposed model is not intended to describe melt-state rheology or behavior far above *T_g_*. Third, the strain rate was controlled using the crosshead displacement speed rather than direct strain measurement. Fourth, the proposed constitutive model is applicable only to the pre-yield regime.

## Figures and Tables

**Table 1:** Constitutive model parameters at different temperatures.

*T* (°C)	*E*_0_ (GPa)	*C*_1_ (GPa)	*C*_2_ (GPa)	*C*_3_ (GPa)	*E*_1_ (MPa)	*θ*_0_ (s)	*β*
20	2.76	1.76	−4.67 × 10^2^	2.82 × 10^3^	2.87 × 10^2^	0.21	0.5
80	2.46	1.25 × 10^1^	−9.36 × 10^2^	5.77 × 10^3^	2.97 × 10^2^	0.30	0.5
110	2.17	1.72 × 10^1^	−1.30 × 10^3^	8.33 × 10^3^	5.25 × 10^2^	0.57	0.5
140	0.85	0.56	3.15 × 10^1^	−3.13 × 10^4^	1.74 × 10^3^	1.23	0.5

**Table 2:** Dimensionless expression of interpolation function.

Model Parameters	Interpolation Functions	*R*^2^ (*cod*)
*E*_0_	*E*_0_(*T*) = 3.22 × 10^3^ − 31.89*T* + 0.536*T*^2^ − 0.0031*T*^3^	0.993
*C*_1_	*C*_1_(*T*) = 1.32 × 10^4^ − 9.02 × 10^2^*T* + 18.44*T*^2^ − 0.09*T*^3^	0.986
*C*_2_	*C*_2_(*T*) = −1.34 × 10^6^ + 6.54 × 10^4^*T* − 1.27 × 10^3^*T*^2^ + 6.24*T*^3^	0.975
*C*_3_	*C*_3_(*T*) = 2.32 × 10^7^ − 1.49 × 10^6^*T* + 2.77 × 10^4^*T*^2^ − 1.42 × 10^2^*T*^3^	0.981
*E*_1_	*E*_1_(*T*) = −5.076 + 22.37*T* − 0.47*T*^2^ + 0.0028*T*^3^	0.996
*θ*_0_	*θ*_0_(*T*) = 0.4047 − 0.0118*T* + 0.00012*T*^2^	0.992

## Data Availability

Data will be made available on request.
